# Supplementary material for: AcaFinder: Genome Mining for Anti-CRISPR-Associated Genes
Source: mSystems. 2022 Nov 22;7(6):e00817-22. doi: 10.1128/msystems.00817-22 (PMC9765179; doi:10.1128/msystems.00817-22)
Supplement: FIG S1 [file msystems.00817-22-s0001.pdf]

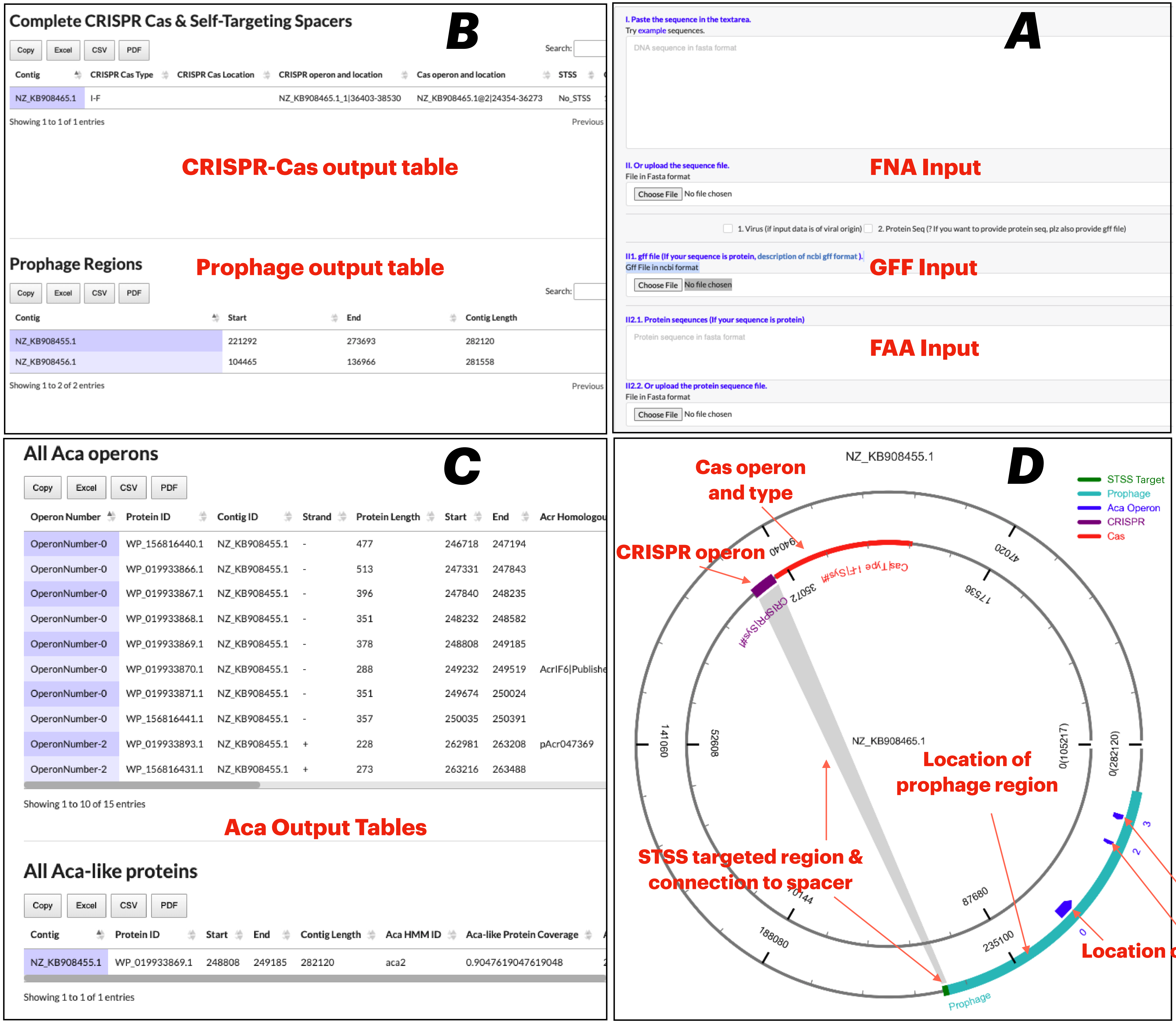
A
I. Paste the sequence in the textarea.
Try example sequences.
DNA sequence in fasta format
FNA Input
II. Or upload the sequence file.
File in Fasta format
Choose File No file chosen
1. Virus (if input data is of viral origin)
2. Protein Seq (? If you want to provide protein seq, plz also provide gff file)
II1. gff file (If your sequence is protein, description of ncbi gff format).
Gff File in ncbi format
GFF Input
Choose File No file chosen
II2.1. Protein seqeunces (If your sequence is protein)
Protein sequence in fasta format
FAA Input
II2.2. Or upload the protein sequence file.
File in Fasta format
Choose File No file chosen
B
Complete CRISPR Cas & Self-Targeting Spacers
Copy Excel CSV PDF
Search:
Contig | CRISPR Cas Type | CRISPR Cas Location | CRISPR operon and location | Cas operon and location | STSS
NZ_KB908465.1 | I-F | | NZ_KB908465.1_1|36403-38530 | NZ_KB908465.1@2|24354-36273 | No_STSS
Showing 1 to 1 of 1 entries
Previous
CRISPR-Cas output table
Prophage Regions
Prophage output table
Copy Excel CSV PDF
Search:
Contig | Start | End | Contig Length
NZ_KB908455.1 | 221292 | 273693 | 282120
NZ_KB908456.1 | 104465 | 136966 | 281558
Showing 1 to 2 of 2 entries
Previous
C
All Aca operons
Copy Excel CSV PDF
Operon Number | Protein ID | Contig ID | Strand | Protein Length | Start | End | Acr Homologou
OperonNumber-0 | WP_156816440.1 | NZ_KB908455.1 | - | 477 | 246718 | 247194 |
OperonNumber-0 | WP_019933866.1 | NZ_KB908455.1 | - | 513 | 247331 | 247843 |
OperonNumber-0 | WP_019933867.1 | NZ_KB908455.1 | - | 396 | 247840 | 248235 |
OperonNumber-0 | WP_019933868.1 | NZ_KB908455.1 | - | 351 | 248232 | 248582 |
OperonNumber-0 | WP_019933869.1 | NZ_KB908455.1 | - | 378 | 248808 | 249185 |
OperonNumber-0 | WP_019933870.1 | NZ_KB908455.1 | - | 288 | 249232 | 249519 | AcrIF6|Publishe
OperonNumber-0 | WP_019933871.1 | NZ_KB908455.1 | - | 351 | 249674 | 250024 |
OperonNumber-0 | WP_156816441.1 | NZ_KB908455.1 | - | 357 | 250035 | 250391 |
OperonNumber-2 | WP_019933893.1 | NZ_KB908455.1 | + | 228 | 262981 | 263208 | pAcr047369
OperonNumber-2 | WP_156816431.1 | NZ_KB908455.1 | + | 273 | 263216 | 263488 |
Showing 1 to 10 of 15 entries
Aca Output Tables
All Aca-like proteins
Copy Excel CSV PDF
Contig | Protein ID | Start | End | Contig Length | Aca HMM ID | Aca-like Protein Coverage
NZ_KB908455.1 | WP_019933869.1 | 248808 | 249185 | 282120 | aca2 | 0.9047619047619048
Showing 1 to 1 of 1 entries
D
NZ_KB908455.1
STSS Target
Prophage
Aca Operon
CRISPR
Cas
Cas operon and type
CRISPR operon
Cas|Type I-F|Sys#
CRISPR|Sys#
NZ_KB908465.1
94040
47020
17536
35072
52608
141060
0(105217)
0(282120)
87680
235100
188080
70144
0
2
3
Prophage
Location of prophage region
STSS targeted region & connection to spacer
Location of Aca operon
