# Supplementary figures and images for: AcaFinder: Genome Mining for Anti-CRISPR-Associated Genes
Source: mSystems. 2022 Nov 22;7(6):e00817-22. doi: 10.1128/msystems.00817-22 (PMC9765179; doi:10.1128/msystems.00817-22)

Tree scale: 1

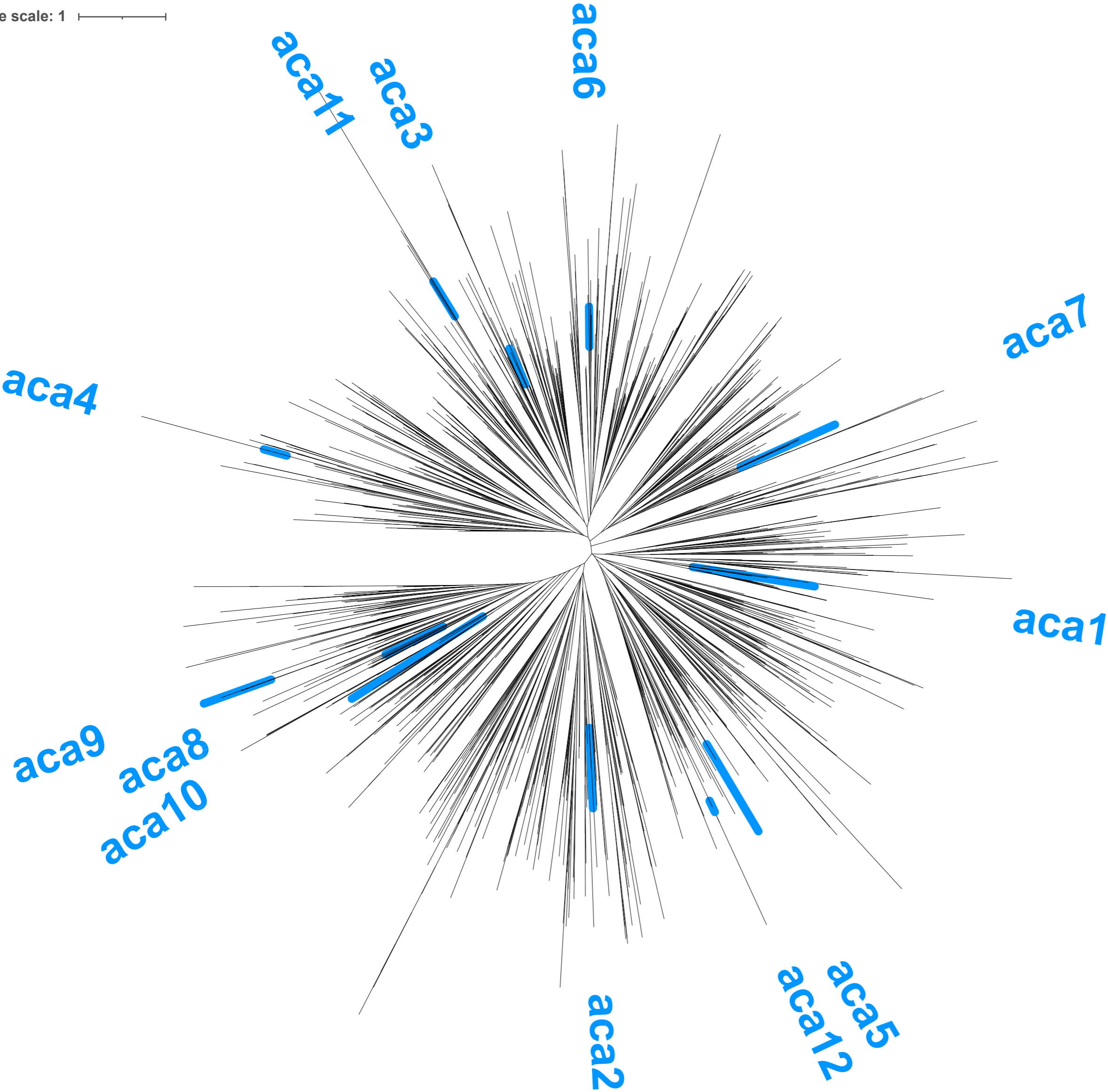

Supplement: FIG S2 [file msystems.00817-22-s0002.pdf]
